# Functional annotation of *de novo* variants from healthy individuals

**DOI:** 10.5808/GI.2019.17.4.e46

**Published:** 2019-12-23

**Authors:** Jean Lee, Sung Eun Hong

**Affiliations:** Department of Biomedical Sciences, Seoul National University College of Medicine, Seoul 03080, Korea

**Keywords:** *de novo* variants, functional annotation, healthy population, whole genome sequencing

## Abstract

The implications of germline *de novo* variants (DNVs) in diseases are well documented. Despite extensive research, inconsistencies between studies remain a challenge, and the distribution and genetic characteristics of DNVs need to be precisely evaluated. To address this issue at the whole-genome scale, a large number of DNVs identified from the whole-genome sequencing of 1,902 healthy trios (i.e., parents and progeny) from the Simons Foundation for Autism Research Initiative study and 20 healthy Korean trios were analyzed. These apparently nonpathogenic DNVs were enriched in functional elements of the genome but relatively depleted in regions of common copy number variants, implying their potential function as triggers of evolution even in healthy groups. No strong mutational hotspots were identified. The pathogenicity of the DNVs was not strongly elevated, reflecting the health status of the cohort. The mutational signatures were consistent with previous studies. This study will serve as a reference for future DNV studies.

## Introduction

*De novo* variants (DNVs) are mutations that are not inherited from parents but arise from mutational events during gametogenesis and embryogenesis. DNVs are believed to be a source of genetic variation at the population scale and can be used for studying evolutionary processes [1]. They are the causal mutations of a variety of diseases [2-5].

Studies using family whole-genome sequencing (WGS) revealed that the average de novo substitution rate per generation ranges from 1.0–1.5 × 10^–8^ per base, resulting in approximately 74 DNVs per person [6]. Many factors affect mutation rates, including extrinsic factors such as parental age at conception and intrinsic factors such as genetic context, GC content and DNA hypersensitivity. However, previous reports have revealed inconsistent DNV rates, which remain to be clarified [7].

The identification of DNVs is challenging because high-coverage WGS data of probands and parents are required for reliable DNV detection. Compared to inherited variants, DNVs are rare and require a large cohort to obtain enough statistical power to detect reliable patterns within DNVs [8]. Furthermore, it is difficult to prove a causal relationship between a DNV and a phenotype because the probability of finding another individual with the same DNV is scarce.

The genetics of autism spectrum disorder has been extensively studied, leading to the identification of many disease-related genes [9-11]. Recently, noncoding regions were actively investigated for associations with autism risk in a large pool of quartet families including one affected child and an unaffected sibling [12]. While the previous study mainly investigated differences between DNVs in the patient group and the control group, we obtained DNVs only from the control group. Along with the WGS results of the healthy Korean trios, we analyzed the distribution and patterns of DNVs in a total of 1,922 healthy individuals. 

## Methods

### Datasets

The trio-based genome data for DNV calling were approved for use and downloaded. The Korean Bioinformation Center (KOBIC) cohort data of 65 individuals (20 families) were downloaded in Variant Call Format from the Genome InfraNet (http://ginet.kr, #10050164) maintained by KOBIC.

The Simons Foundation for Autism Research Initiative (SFARI) cohort data were obtained from Supplementary Table 2 of a previously published article [12] in which WGS was performed with a mean coverage of 35.5× in 1,902 autism spectrum disorder quartet families (1 affected child, 1 healthy sibling and their parents). Data from healthy siblings were used for further analysis. Since the sequencing data were hosted by SFARI, the variants from this list will be designated as DNVs from SFARI.

### DNVs from the KOBIC database and SFARI

A total of 15 trio and 5 quartet families (total of 65 individuals) were identified from the KOBIC cohort. From the quartet samples, only one sibling was included for further analysis. Variants that were present in the probands and were not present in both parents were selected. The following filtering criteria were used: QUAL > 200, DP > 20 and custom-defined GQ values. Annotation was performed with Variant Effect Predictor [13]. Variants exhibiting segmental duplication (SEGDUP) and or an LCR flag (low complexity region) were excluded in KOBIC cohort. Overlapping variants between individuals in our cohorts and variants that were already reported in gnomAD [14] were excluded. DNVs from SFARI cohort were used for downstream analysis without any additional filtering.

### Downstream analyses

The mutational spectrum of the DNVs and the contribution of 30 well-known COSMIC [15] mutational signatures were calculated by using MuSiCa [16]. The distribution of DNVs was plotted with karyoPlotR [17]. The enrichment of DNVs in different genome regions of genomes was evaluated with GAT [18]. BED files containing the coordinates of the 3′-untranslated region (UTRs), 5′-UTRs, exons, and introns were obtained from the University of California Santa Cruz (UCSC) Table Browser [19]. A comprehensive gene annotation file for the whole genome (GRCh 38) was downloaded from GENCODE (version 32) [20], and regions with no genic annotations were extracted as intergenic regions. Regions of common structural variants were obtained from the gnomAD version 2 structural variants [14]. Variants classified as duplication, deletion, or multiallelic copy number variation (MCNV) were selected, and alleles with a frequency (maximum value for MCNV) exceeding 1% or 5% were selected, transformed according to hg38, and used for further study.

### CADD score calculation

Variants in the gnomAD [14] version 2 exome with an allele frequency >1% were selected. Variants in ClinVar [21] were downloaded, and those for which the clinical significance denoted as “pathogenic” or “likely pathogenic” were selected. The raw unscaled CADD scores [22] of DNVs from KOBIC, SFARI, common gnomAD single nucleotide polymorphisms (SNPs), and pathogenic ClinVar SNPs were calculated.

## Results and Discussion

**Fig. 1.**
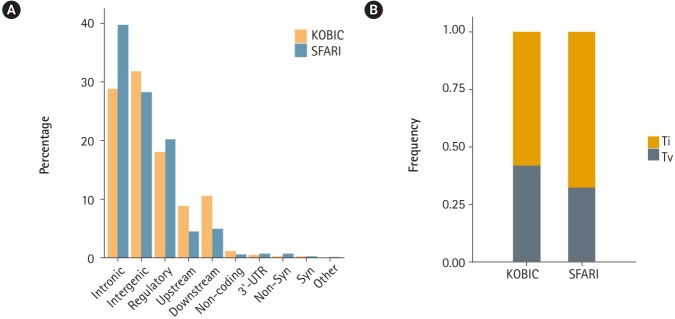
Profile of *de novo* variants (DNVs) in the Simons Foundation for Autism Research Initiative (SFARI) and Korean Bioinformation Center (KOBIC) cohorts. (A) DNV profile by genomic position. 5′-untranslated region (UTR), canonical splice, splice, stop gain, and stop lost variants are classified as “others.” (B) Ratios of transitions (Ti) and transversions (Tv) in each cohort.

A total of 455 and 115,870 DNVs were called from the KOBIC and SFARI cohorts, respectively. Most of the DNVs were located in intronic and intergenic regions (Fig. 1A), as these regions encompass the majority of the genome (~96.5% [23]). The transition to transversion ratio of the DNVs was 2.1 in the SFARI cohort (Fig. 1B), which was within the expected range of 2.0–2.1 [24]. However, the variants from the KOBIC cohort displayed a ratio of 1.4, implying undercalling of transition variants. Between the two types of transition substitutions, C>T changes were 1.4 times more abundant than T>C variants after correcting for the base composition in the genome. C>T substitutions were 1.9 times more frequent in the CpG dinucleotide context than in the non-CpG dinucleotide context after correcting for the base composition. This result suggests hypermutability of CpG dinucleotides in which methylated cytosine undergoes deamination, leading to more frequent C>T changes [25,26].

**Fig. 2.**
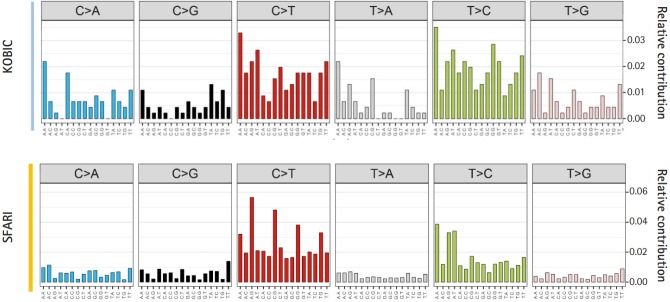
Mutational signature of *de novo* variants (DNVs). Each DNV change is plotted according to the sequences including one base before and after each DNV. KOBIC, Korean Bioinformation Center; SFARI, Simons Foundation for Autism Research Initiative.

The sequence context of DNVs shapes mutation rates. The mutational signature was originally used for the modeling of mutational processes in a somatic mutation analysis of cancers [27,28], which is widely used in various mutational analyses. Therefore, we surveyed the sequence contexts of our DNVs. While the mutational spectrum of DNVs from SFARI closely resembled the previously reported mutational spectrum of germline DNVs (Fig. 2) [29], the DNVs from KOBIC showed slight differences, implying a need for a larger sample size and further verification of DNV calls.

Additionally, we reconstructed our mutational spectrum with 30 well-known signatures curated by COSMIC and quantified the contribution of each signature. Signatures 1, 5, and 16 contributed the majority of the signatures, contributing 32%, 25% and 31% of the total, respectively. These findings are consistent with a previous report [29] that demonstrated that signatures 1 and 5 explained most of the observed germline DNVs. Signature 1 represents spontaneous deamination of methylated cytosine and the subsequent mutational process. No proposed etiology is suggested for signatures 5 and 16, but both exhibit strand bias during transcription in T>C variants in the trinucleotide context of ApTpN.

**Fig. 3.**
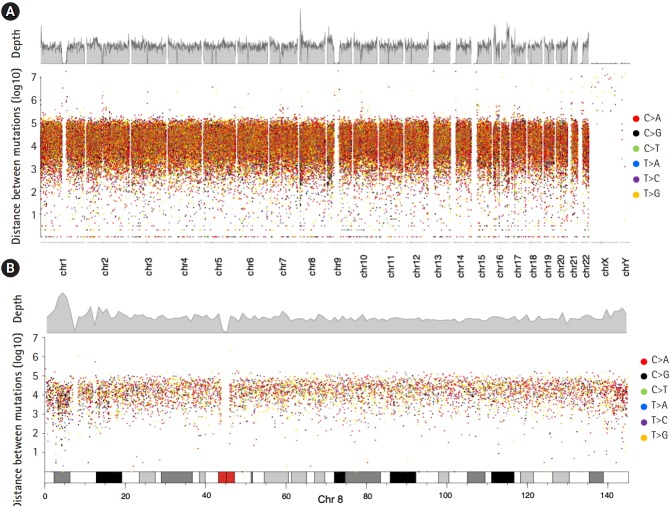
Rainfall plot of *de novo* variants (DNVs). (A) Genome-wide plot displaying all DNVs. Top, density of DNVs with a window width of 106 bases. Bottom, rainfall plot of germline DNVs by chromosome. (B) Rainfall plot in chromosome 8. The bottom bar represents the karyotype structure of chromosome 8, and the red box indicates a centromeric region.

A rainfall plot was employed to visualize mutational hotspots [30], and our DNVs did not display a strong signature of mutational hotspots (Fig. 3).

**Fig. 4.**
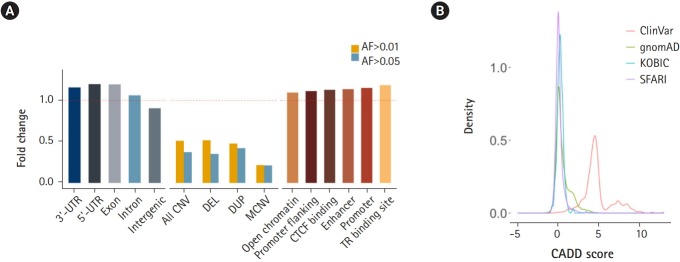
Enrichment of *de novo* variants (DNVs) by functional annotation. (A) Distribution of DNVs in genic regions and intergenic regions (left), in common copy number variation (CNV) regions (middle), and grouped by regulatory elements. (B) CADD score distribution of DNVs. UTR, untranslated region; DEL, deletion; DUP, duplication; MCNV, multiallelic CNV; AF, allele frequency; KOBIC, Korean Bioinformation Center; SFARI, Simons Foundation for Autism Research Initiative.

Next, we subjected various annotated genetic elements to DNV burden analysis. Genic regions including 3′-UTRs, 5′-UTRs, exons and introns were enriched, as shown by the ratio of the observed count to the expected counts exceeding 1.0. In contrast, intergenic regions were depleted of DNVs (Fig. 4A). Regions with a high copy number variation (CNV) frequency (allele frequency > 0.01 or 0.05) were tested for DNV enrichment. These regions were depleted of DNVs, and regions with higher allele frequency (0.05) and multiallelic regions exhibited greater depletion. This result suggests that DNVs tend to occur in regions that are thought to be less tolerant of copy number changes (Fig. 4A). Approximately half of high-frequency CNV regions were SEGDUP regions, which were depleted with DNVs (37% of expected) to a similar degree as high-frequency CNV regions. Since SEGDDUP regions are vulnerable to undercalling, such impact requires further study. The enrichment of DNVs in functional regions and their depletion in less-functional regions imply the potential roles of DNVs in generating new functional alleles, resulting in the incorporation of new alleles into a population. Additionally, all six regulatory elements were enriched with DNVs in our cohort (Fig. 4A).

The prediction of DNV pathogenicity quantified by the CADD score [22] showed a similar distribution pattern to common SNPs [14], while pathogenic variants from the ClinVar [21] database showed higher scores (Fig. 4B). Finally, we surveyed our 116,325 DNVs against the ClinVar [21] database to check whether there are cryptic DNVs that may be associated with diseases. Five variants were enlisted in the ClinVar database as pathogenic or likely pathogenic [21]. Only one variant in *RAPSN* (p.Val45Met), which is known to cause myasthenic syndrome with an autosomal recessive pattern, was functionally assayed [31-33]. Although DNVs frequently occur in the functional elements of the genome, the CADD score [22] distribution resembling that of nonpathogenic variants and the lack of pathogenic variants reflect the health status of the cohorts.

Here, we analyzed the distribution and genetic patterns of 116,325 DNVs derived from 1,922 healthy individuals. The mutational signatures were consistent with previously reported signatures. We could not identify strong mutation hotspots in our cohort. Notably, the DNVs were enriched within elements with potential functionality, such as genic regions and regulatory regions, but depleted in intergenic regions and regions that are tolerant to copy number changes. This observation was unexpected since the carriers of these DNVs are healthy and are not expected to display enrichment in functional regions of the genome. This enrichment was not strong enough to be differentiated by the CADD scores. Due to the discrepancies in the sizes of the cohorts (1,902 for SFARI and 20 for KOBIC), their ethnicity compositions and the sequencing procedures applied, direct comparison between the two databases is challenging. However, regarding the consistency of the mutational spectrum and the signatures of the SFARI DNVs with previous studies, the DNVs from KOBIC are expected to follow the patterns of the DNVs from SFARI with a larger cohort size and DNV validation. Further research involving epigenetic signatures and individualized mutational cluster analysis may elucidate the factors affecting the germline mutation rate, leading to better identification of disease-associated DNVs and an improved understanding of human genome evolution.
